# Supplementary material for: Early Stroke Induces Long-Term Impairment of Adult Neurogenesis Accompanied by Hippocampal-Mediated Cognitive Decline
Source: Cells. 2019 Dec 17;8(12):1654. doi: 10.3390/cells8121654 (PMC6953020; doi:10.3390/cells8121654)
Supplement: Supplementary file 1 [file cells-08-01654-s001.zip › cells-629059-supplementary-final/Neuer Ordner/Kathner-Schaffert_S1_antibodies.pdf]

Supplement S1

Table: Primary antibodies and secondary antibodies

Primary antibody	Dilution	Source
mouse anti MAP2	1 : 10000	Sigma-Aldrich, USA
rabbit anti Ki67	1 : 400	Leica, Wetzlar, Germany
rat anti BrdU	1 : 500	AbD Serotec, Kidlington, UK
mouse anti NeuN	1 : 500	Chemicon; Billerica, USA
goat anti GFP	1 : 500	Acris, Germany
guinea pig anti DCX	1 : 500	Millipore, UK
rabbit anti GFAP	1 : 500	Synaptic Systems, Germany
guinea pig anti GFAP	1 : 500	Zytomed Systems, Berlin, Germany
mouse anti PCNA	1 : 500	Signet, USA
rabbit anti RFP	1 : 500	Abcam, UK

Secondary antibody	Dilution	Source
IgG biotinylated donkey anti rabbit	1 : 500	Dianova, Hamburg, Germany
IgG biotinylated donkey anti rat	1 : 500	Dianova, Hamburg, Germany
IgG biotinylated donkey anti mouse	1 : 500	Dianova, Hamburg, Germany
Rhodamine anti-rat	1 : 500	Dianova, Hamburg, Germany
Rhodamine anti-mouse	1 : 500	Dianova, Hamburg, Germany
Rhodamine anti-rabbit	1 : 500	Dianova, Hamburg, Germany
Alexa Fluor 488 anti-goat	1 : 500	Invitrogen, Carlsbad, CA
Cy5 anti-guinea pig	1 : 500	Dianova, Hamburg, Germany
Cy5 anti-mouse	1 : 500	Dianova, Hamburg, Germany
