# Supplementary material for: Early Stroke Induces Long-Term Impairment of Adult Neurogenesis Accompanied by Hippocampal-Mediated Cognitive Decline
Source: Cells. 2019 Dec 17;8(12):1654. doi: 10.3390/cells8121654 (PMC6953020; doi:10.3390/cells8121654)
Supplement: Supplementary file 1 [file cells-08-01654-s001.zip › cells-629059-supplementary-final/Neuer Ordner/Kathner-Schaffert_S4_Sholl analysis.pdf]

Supplemental S4

Table: Parameters estimate for Sholl analysis of retroviral vector labelled neurons

	MCAO			Sham				
**Radius (μm)**	**Intersections**	**±**	**SEM**	**Intersections**	**±**	**SEM**	**F-value**	**p-value**
10	1.167	±	0.18	1.286	±	0.17	F(1)=0.223	p=0.646
15	1.333	±	0.37	1.42	±	0.37	F(1)=0.064	p=0.805
20	2.16	±	0.36	1.42	±	0.33	F(1)=2.265	p=0.160
25	2.16	±	0.43	1.57	±	0.40	F(1)=1.004	p=0.338
30	2.33	±	1.43	2.86	±	0.39	F(1)=0.800	p=0.390
35	2.50	±	0.45	3.00	±	0.41	F(1)=0.658	p=0.434
40	2.50	±	0.45	3.00	±	0.41	F(1)=0.658	p=0.434
45	3.17	±	0.35	4.28	±	0.32	F(1)=5.387	**p=0.041**
50	3.00	±	0.45	4.71	±	0.41	F(1)=7.77	p=0.018
55	3.67	±	0.55	4.86	±	0.51	F(1)=2.495	p=0.143
60	3.67	±	0.56	4.43	±	0.52	F(1)=0.98	p=0.343
65	4.00	±	0.57	4.71	±	0.52	F(1)=0.846	p=0.377
70	3.83	±	0.51	4.86	±	0.47	F(1)=2.106	p=0.175
75	4.17	±	0.55	4.71	±	0.51	F(1)=0.526	p=0.483
80	3.83	±	0.46	4.57	±	0.43	F(1)=1.331	p=0.273
85	3.83	±	0.46	4.57	±	0.43	F(1)=1.331	p=0.273
90	3.50	±	0.41	5.00	±	0.38	F(1)=6.95	**p=0.023**
100	4.17	±	0.38	4.86	±	0.35	F(1)=1.748	p=0.213
105	4.17	±	0.44	5.00	±	0.40	F(1)=1.923	p=0.193
110	4.33	±	0.48	5.00	±	0.44	F(1)=1.030	p=0.332
115	4.50	±	0.60	4.86	±	0.56	F(1)=0.186	p=0.675
120	4.50	±	0.56	4.57	±	0.52	F(1)=0.009	p=0.928
125	5.17	±	0.63	4.57	±	0.58	F(1)=0.474	p=0.505
130	4.83	±	0.61	4.57	±	0.56	F(1)=0.099	p=0.759
135	4.50	±	0.61	4.57	±	0.57	F(1)=0.007	p=0.934

140	4.50 ± 0.71	5.00 ± 0.66	F(1)=0.265	p=0.617
145	4.33 ± 0.73	5.00 ± 0.67	F(1)=0.447	p=0.518
150	4.33 ± 0.76	4.86 ± 0.70	F(1)=0.255	p=0.623
155	4.83 ± 0.92	5.29 ± 0.85	F(1)=0.129	p=0.726
160	4.50 ± 0.94	5.43 ± 0.87	F(1)=0.517	p=0.487
165	4.33 ± 1.03	5.57 ± 0.96	F(1)=0.767	p=0.40
170	4.00 ± 0.76	5.83 ± 0.76	F(1)=2.895	p=0.12
175	3.67 ± 0.58	5.17 ± 0.58	F(1)=3.347	p=0.097
180	3.50 ± 0.58	5.17 ± 0.58	F(1)=4.098	p=0.070
185	3.17 ± 0.70	4.67 ± 0.70	F(1)=2.238	p=0.166
190	3.00 ± 0.71	4.17 ± 0.71	F(1)=1.324	p=0.277
195	3.00 ± 0.77	4.00 ± 0.77	F(1)=0.833	p=0.383
200	3.00 ± 0.74	3.17 ± 0.74	F(1)=0.025	p=0.877
205	3.00 ± 0.76	3.33 ± 0.76	F(1)=0.094	p=0.765
210	3.00 ± 0.59	2.50 ± 0.59	F(1)=0.349	p=0.568
215	3.00 ± 0.48	2.00 ± 0.48	F(1)=2.143	p=0.174
220	2.83 ± 0.45	1.33 ± 0.45	F(1)=5.548	**p=0.040**
225	2.50 ± 0.56	1.50 ± 0.56	F(1)=0.579	p=0.237
230	2.17 ± 0.26	0.667 ± 0.26	F(1)=16.20	**p=0.002**

Statistical differences of intersections between the groups are validated by one-way ANOVA (dependent factors: days; factors: group).
