# Supplementary material for: Early Stroke Induces Long-Term Impairment of Adult Neurogenesis Accompanied by Hippocampal-Mediated Cognitive Decline
Source: Cells. 2019 Dec 17;8(12):1654. doi: 10.3390/cells8121654 (PMC6953020; doi:10.3390/cells8121654)
Supplement: Supplementary file 1 [file cells-08-01654-s001.zip › cells-629059-supplementary-final/Neuer Ordner/Kathner-Schaffert_S7_Hippocampus-dependent strategies.pdf]

Supplement S7

Table: Percentage usage of hippocampal-dependent strategies

Groups	day1		day2		day3		day4		day5	
	**MW**	**SEM**	**MW**	**SEM**	**MW**	**SEM**	**MW**	**SEM**	**MW**	**SEM**
6 month MCAO	5,67	3,58	2,83	2,83	47,00	16,83	27,67	6,98	31,67	8,09
6 month sham	33,30	7,87	45,60	9,49	76,90	6,46	27,00	8,95	66,80	6,05
7.5 month MCAO	19,50	7,93	25,00	11,18	50,00	9,58	8,50	3,80	5,67	3,58
7.5 month sham	22,88	9,89	54,25	5,38	70,75	5,83	29,25	6,68	74,88	5,20
9 month MCAO	29,00	10,46	29,25	7,88	46,00	17,06	37,50	10,53	37,50	10,53
9 month sham	16,80	9,13	43,40	12,55	66,40	10,51	30,00	13,38	60,00	6,63
20 month MCAO	26,92	7,17	33,23	4,62	49,38	8,98	25,69	5,85	48,72	7,17
20 month sham	31,50	2,94	30,10	9,25	43,40	8,57	30,20	8,17	58,20	6,70
