# Supplementary material for: Early Stroke Induces Long-Term Impairment of Adult Neurogenesis Accompanied by Hippocampal-Mediated Cognitive Decline
Source: Cells. 2019 Dec 17;8(12):1654. doi: 10.3390/cells8121654 (PMC6953020; doi:10.3390/cells8121654)
Supplement: Supplementary file 1 [file cells-08-01654-s001.zip › cells-629059-supplementary-final/Neuer Ordner/Kathner_Schaffert_S2_ Volume_statistic.pdf]

Supplement S2: Brain, lesion, hippocampal and dentate gyrus volumes

Statistical differences between sham and MCAO groups

Brain volume

6 month group:
sham Mdn = 203 mm^3; IqR = 38.50
MCAO Mdn = 194 mm^3; IqR = 33.50; U = 29.50; n = 16; p = 0.235

7.5 month group:
sham Mdn = 192 mm^3; IqR = 30.50
MCAO Mdn = 192 mm^3; IqR = 44.00; U = 41.00; n = 16; p = 0.780

9 month group:
sham Mdn = 215 mm^3; IqR = 21.75
MCAO Mdn = 143 mm^3; IqR = 74.00; U = 6.00; n = 12 ; p = 0.109

20 month group:
sham Mdn = 204 mm^3; IqR = 9.91
MCAO Mdn = 195 mm^3; IqR = 16.86; U = 25.00; n = 17; p = 0.113

Hippocampal volume

6 month group:
sham Mdn = 6.1 mm^3; IqR = 22.50
MCAO Mdn = 5.2 mm^3; IqR = 16.00; U = 6.50; n = 10; p = 0.222

7.5 month group:
sham Mdn = 5.4 mm^3; IqR = 21.25
MCAO Mdn = 5.1 mm^3; IqR = 19.25; U = 8.00; n = 11; p = 0.247

9 month group:
sham Mdn = 5.7 mm^3; IqR = 13.25
MCAO Mdn = 3.5 mm^3; IqR = 28.25; U = 4.00; n = 10; p = 0.114

Dentate gyrus volume

6 month group:
sham Mdn = 0.67 mm^3; IqR = 0.18
MCAO Mdn = 0.65 mm^3; IqR = 0.13; U = 27.00; n = 16; p = 0.500

7.5 month group:
sham Mdn = 0.55 mm^3; IqR = 0.13
MCAO Mdn = 0.72 mm^3; IqR = 0.39; U = 11.00; n = 13; p = 0.222

9 month group:

sham Mdn = 0.58 mm^3; IqR = 0.05
MCAO Mdn = 0.45 mm^3; IqR = 0.28; U = 8.00; n = 12; p = 0.214

20 month group:
sham Mdn = 0.60 mm^3; IqR = 0.07
MCAO Mdn = 0.62 mm^3; IqR = 0.17; U = 6.00; n = 10; p = 0.172

Statistical differences within the MCAO groups

Brain volume

6m *versus* 7.5m:	U = 25.50, n = 15, p = 0.635
6m *versus* 9m:	U = 6.00, n = 10, p = 0.257
7.5m *versus* 9m:	U = 6.00, n = 10, p = 0.257
6m *versus* 20m:	U = 23.00, n = 16, p = 0.492
7.5m *versus* 20m:	U = 45.00, n = 20, p = 0.739
9m *versus* 20m:	U = 10.00, n = 14, p = 0.188

Lesion volume

6 month group:	Mdn = 2.9 mm^3; IqR = 1.25
7.5 month group:	Mdn = 2.1 mm^3; IqR = 0.50
9 month group:	Mdn = 2.4 mm^3; IqR = 1.00
20 month group:	Mdn = 1.89 m^3; IqR = 0.82

6m *versus* 7.5m:	U = 18.00, n = 15, p = 0.220
7.5m *versus* 9m:	U = 15.00, n = 10, p = 0.539
6m *versus* 9m:	U = 9.00, n = 10, p = 0.610
6m *versus* 20m:	U = 4.00, n = 12, p = 0.024
7.5m *versus* 20m:	U = 20.00, n = 16, p = 0.313
9m *versus* 20m:	U = 20.00, n = 16, p = 0.257

Statistical differences within the sham groups

Brain volume

6m *versus* 7.5m:	U = 56.00, n = 24, p = 0.519
7.5m *versus* 9m:	U = 6.50, n = 17, p = 0.002
6m *versus* 9m:	U = 25.00, n = 23, p = 0.024
6m *versus* 20m:	U = 65.00, n = 24, p = 0.907
7.5m *versus* 20m:	U = 29.00, n = 18, p = 0.340
9m *versus* 20m:	U = 7.00, n = 17, p = 0.004
