# Supplementary material for: Early Stroke Induces Long-Term Impairment of Adult Neurogenesis Accompanied by Hippocampal-Mediated Cognitive Decline
Source: Cells. 2019 Dec 17;8(12):1654. doi: 10.3390/cells8121654 (PMC6953020; doi:10.3390/cells8121654)
Supplement: Supplementary file 1 [file cells-08-01654-s001.zip › cells-629059-supplementary-final/Neuer Ordner/Kathner_Schaffert_S3_ Ki67_neurogenesis_statistic.pdf]

Supplement S4: Ki67-positive cells and neurogenesis

Ki67-positive cells

Statistical differences within sham groups

sham groups:

6m *versus* 7.5m:	$U = 3.00$; $n = 11$; $p = 0.028$
6m *versus* 9m:	$U < 0.001$; $n = 10$; $p = 0.009$
7.5m *versus* 9m:	$U < 0.001$; $n = 10$; $p = 0.009$

Statistical differences within MCAO groups

MCAO groups:

6m *versus* 7.5m:	$U = 5.00$; $n = 12$; $p = 0.042$
6m *versus* 9m:	$U < 0.001$; $n = 13$; $p = 0.003$
7.5m *versus* 9m:	$U = 3.00$; $n = 11$; $p = 0.009$

Statistical differences between sham and MCAO

6 month groups:

Sham:	Mdn = 588 cells; IqR = 348
MCAO:	Mdn = 606 cells; IqR = 285; $U = 17.50$; $n = 13$; $p = 0.617$

7.5 month groups:

Sham:	Mdn = 432 cells; IqR = 114
MCAO:	Mdn = 372 cells; IqR = 120; $U = 5.00$; $n = 10$; $p = 0.112$

9 month groups:

Sham:	Mdn = 300 cells; IqR = 78.00
MCAO:	Mdn = 132 cells; IqR = 72.00; $U < 0.001$; $n = 11$; $p = 0.006$

Adult neurogenesis

Statistical differences within sham groups

sham groups:

6m *versus* 7.5m:	$U = 17.00$; $n = 14$; $p = 0.383$
6m *versus* 9m:	$U = 21.00$; $n = 13$; $p = 1.000$
7.5m *versus* 9m:	$U = 10.00$; $n = 13$; $p = 0.138$
6m *versus* 20m:	$U < 0.001$; $n = 13$; $p = 0.001$
7.5m *versus* 20m:	$U < 0.001$; $n = 13$; $p = 0.001$
9m *versus* 20m:	$U < 0.001$; $n = 12$; $p = 0.002$

Statistical differences within MCAO groups

6m *versus* 7.5m:	$U = 17.00$; $n = 14$; $p = 0.016$
6m *versus* 9m:	$U = 21.00$; $n = 13$; $p = 0.111$
7.5m *versus* 9m:	$U = 10.00$; $n = 13$; $p = 0.556$
6m *versus* 20m:	$U < 0.001$; $n = 12$; $p = 0.003$
7.5m *versus* 20m:	$U < 0.001$; $n = 12$; $p = 0.003$
9m *versus* 20m:	$U < 0.001$; $n = 11$; $p = 0.006$

Statistical differences between sham and MCAO

6 month group:	
Sham:	Mdn = 1614 cells; IqR = 927
MCAO:	Mdn = 1270 cells; IqR = 924; $U = 12.50$; $n = 12$; $p = 0.432$
7.5 month group:	
Sham:	Mdn = 1492 cells; IqR = 750
MCAO:	Mdn = 801 cells; IqR = 374; $U = 1.00$; $n = 12$; $p = 0.005$
9 month group:	
Sham:	Mdn = 1604 cells; IqR = 680
MCAO:	Mdn = 1026 cells; IqR = 830; $U = 1.00$; $n = 10$; $p = 0.019$
20-month-old group:	
Sham:	Mdn = 13 cells; IqR = 28.13
MCAO:	Mdn = 28 cells; 37.50; $U = 15.00$; $n = 13$; $p = 0.445$
