# Supplementary material for: Early Stroke Induces Long-Term Impairment of Adult Neurogenesis Accompanied by Hippocampal-Mediated Cognitive Decline
Source: Cells. 2019 Dec 17;8(12):1654. doi: 10.3390/cells8121654 (PMC6953020; doi:10.3390/cells8121654)
Supplement: Supplementary file 1 [file cells-08-01654-s001.zip › cells-629059-supplementary-final/Neuer Ordner/Kathner_Schaffert_S5_ Precursor_PCNAcells_branched_unbranched stem cells_Statistic.pdf]

Supplement S5

Total number of precursor cells

Statistical differences between sham and MCAO

type 1 cells:
sham: Mdn = 1800 cells; IqR = 1404
MCAO: Mdn = 2928 cells; IqR = 1728; U = 12; n = 13; p = 0.242

type 2a cells:
sham: Mdn = 624 cells; IqR = 540
MCAO: Mdn = 768 cells; IqR = 516; U = 13.00; n = 11; p = 0.715

type 2b cells:
sham: Mdn = 168 cells; IqR = 180
MCAO: Mdn = 120 cells; IqR = 162; U = 7.5; n = 11; p = 0.169

type 3 cells:
sham: Mdn = 240 cells; IqR = 228
MCAO: Mdn = 132 cells; IqR = 78; U = 11.00; n = 11; p = 0.460

immature cells:
sham: Mdn = 240 cells; IqR = 204
MCAO: Mdn = 72 cells; IqR = 90; U = 2.50; n = 11; p = 0.022

Total number of PCNA-positive cells

Statistical differences between sham and MCAO

type 1 cells:
sham: Mdn = 12 cells; IqR = 48
MCAO: Mdn = 42 cells; IqR = 21; U = 7.00; n = 9; p = 0.453

type 2a cells:
sham: Mdn = 192 cells; IqR = 204
MCAO: Mdn = 144 cells; IqR = 93; U = 8.00; n = 9; p = 0.623

type 2b cells:
sham: Mdn = 24 cells; IqR = 18
MCAO: Mdn = 42 cells; IqR = 75; U = 6.00; n = 9; p = 0.317

type 3 cells:
sham: Mdn = 24 cells; IqR = 18
MCAO: Mdn = 0 cells; IqR = 18; U = 5.00; n = 11; p = 0.180

Distribution of branched- and unbranched cells

Statistical differences between sham and MCAO

branched stem cells:
sham: 65 ± 12 %
MCAO Mdn = 69 ± 14 %; U = 16.50; n = 13; p = 0.607

unbranched stem cells:
sham 35 ± 12 %
MCAO Mdn = 31 ± 14 %; U = 16.50; n = 13; p = 0.607
