# Supplementary material for: Early Stroke Induces Long-Term Impairment of Adult Neurogenesis Accompanied by Hippocampal-Mediated Cognitive Decline
Source: Cells. 2019 Dec 17;8(12):1654. doi: 10.3390/cells8121654 (PMC6953020; doi:10.3390/cells8121654)
Supplement: Supplementary file 1 [file cells-08-01654-s001.zip › cells-629059-supplementary-final/Neuer Ordner/Kathner_Schaffert_S6_WaterMaze_statistic.pdf]

Supplement S6: WaterMaze data

Water Maze distance, latency and velocity:

Statistical differences within sham groups

Distance:	p = 0.148, F(3) = 1.958
6m *versus* 7.5m:	p = 1.000
6m *versus* 9m:	p = 0.302
6m *versus* 20m:	p = 1.000
7.5m *versus* 9m:	p = 0.427
7.5m *versus* 20m:	p = 1.000
9m *versus* 20m:	p = 0.304
Latency:	p = 0.037; F(3) = 3.337
6m *versus* 7.5m:	p = 1.000
6m *versus* 9m:	p = 0.237
6m *versus* 20m:	p = 1.000
7.5m *versus* 9m:	p = 0.371
7.5m *versus* 20m:	p = 1.000
9m *versus* 20m:	p = 0.031
Velocity:	p < 0.001, F(3) = 45.72;
6m *versus* 7.5m:	p = 1.000;
6m *versus* 9m:	p < 0.001;
6m *versus* 20m	p < 0.001;
7.5m *versus* 9m:	p < 0.001;
7.5m *versus* 20m:	p < 0.001;
9m *versus* 20m:	p < 0.001;

Statistical differences within MCAO groups

Distance:	p < 0.001; F(3) = 21.069
6m *versus* 7.5m:	p = 1.000
6m *versus* 9m:	p < 0.001
6m *versus* 20m:	p = 0.001
7.5m *versus* 9m:	p < 0.001
7.5m *versus* 20m:	p < 0.001
9m *versus* 20m:	p = 0.232
Latency:	p = 0.065; F(3) = 2.840
6m *versus* 7.5m:	p = 1.000
6m *versus* 9m:	p = 1.000
6m *versus* 20m:	p = 0.203
7.5m *versus* 9m:	p = 1.000
7.5m *versus* 20m:	p = 0.131
9m *versus* 20m:	p = 1.000
Velocity:	p < 0.001, F(3) = 16.654;
6m *versus* 7.5m:	p = 1.000;
6m *versus* 9m:	p = 0.001;
6m *versus* 20m:	p = 1.000;
7.5m *versus* 9m:	p < 0.001;
7.5 m *versus* 20m:	p = 1.000;

9m *versus* 20m:	$p < 0.001$;

Statistical differences between sham and MCAO

Distance

6m:	$p < 0.001$; $F(1) = 31.635$
7.5m:	$p < 0.001$; $F(1) = 39.212$
9m:	$p = 0.341$; $F(1) = 1.024$
20m:	$p = 0.179$; $F(1) = 2.016$

Latency

6m:	$p < 0.001$; $F(1) = 30.755$
7.5m:	$p = 0.004$; $F(1) = 13.188$
9m:	$p = 0.584$; $F(1) = 0.326$
20m:	$p = 0.105$; $F(1) = 3.029$

Velocity

6m:	$p = 0.227$, $F(1) = 1.657$;
7.5m:	$p = 0.005$, $F(1) = 12.00$;
9m:	$p = 0.707$, $F(1) = 0.152$;
20m:	$p = 0.106$, $F(1) = 3.013$

Statistical differences between sham and MCAO

Distance:

Day1

6m MCAO *versus* sham:	$p = 1.000$; $F(1) = 3.216$
7.5m MCAO *versus* sham:	$p = 0.599$; $F(1) = 0.293$
9m MCAO *versus* sham:	$p = 0.018$; $F(1) = 8.834$
20m MCAO *versus* sham:	$p = 0.503$; $F(1) = 0.467$

Day2

6m MCAO *versus* sham:	$p < 0.001$; $F(1) = 77.833$
7.5m MCAO *versus* sham:	$p = 0.042$; $F(1) = 5.292$
9m MCAO *versus* sham:	$p = 0.19$; $F(1) = 2.05$
20m MCAO *versus* sham:	$p = 0.543$; $F(1) = 0.382$

Day3

6m MCAO *versus* sham:	$p = 0.008$; $F(1) = 10.246$
7.5m MCAO *versus* sham:	$p = 0.046$; $F(1) = 5.05$
9m MCAO *versus* sham:	$p = 0.139$; $F(1) = 2.695$
20m MCAO *versus* sham:	$p = 0.788$; $F(1) = 0.075$

Day4

6m MCAO *versus* sham:	$p = 0.123$; $F(1) = 2.752$
7.5m MCAO *versus* sham:	$p < 0.001$; $F(1) = 29.856$
9m MCAO *versus* sham:	$p = 0.445$; $F(1) = 0.645$
20m MCAO *versus* sham:	$p = 0.573$; $F(1) = 0.329$

Day5

6m MCAO *versus* sham:	$p = 0.096$ $F(1) = 3.371$
7.5m MCAO *versus* sham:	$p < 0.001$; $F(1) = 78.973$
9m MCAO *versus* sham:	$p = 0.032$; $F(1) = 6.737$
20m MCAO *versus* sham:	$p = 0.237$; $F(1) = 1.497$

Latency:

Day1

6m MCAO *versus* sham:	$p = 0.030$; $F(1) = 6.174$
7.5m MCAO *versus* sham:	$p = 0.922$; $F(1) = 0.01$
9m MCAO *versus* sham:	$p = 0.421$; $F(1) = 0.72$
20m MCAO *versus* sham:	$p = 0.175$; $F(1) = 1.993$

Day2

6m MCAO *versus* sham:	$p < 0.001$; $F(1) = 60.429$
7.5m MCAO *versus* sham:	$p = 0.169$; $F(1) = 2.169$
9m MCAO *versus* sham:	$p = 0.666$; $F(1) = 0.201$
20m MCAO *versus* sham:	$p = 0.643$; $F(1) = 0.221$

Day3

6m MCAO *versus* sham:	$p = 0.127$; $F(1) = 2.692$
7.5m MCAO *versus* sham:	$p = 0.119$; $F(1) = 2.864$
9m MCAO *versus* sham:	$p = 0.366$; $F(1) = 0.917$
20m MCAO *versus* sham:	$p = 0.785$; $F(1) = 0.077$

Day4

6m MCAO versus sham:	$p = 0.872$; $F(1) = 0.027$
7.5m MCAO versus sham:	$p = 0.004$; $F(1) = 13.639$
9m MCAO versus sham:	$p = 0.958$; $F(1) = 0.003$
20m MCAO versus sham:	$p = 0.221$; $F(1) = 1.593$

Day5

6m MCAO versus sham:	$p = 0.124$; $F(1) = 2.817$
7.5m MCAO versus sham:	$p < 0.001$; $F(1) = 38.874$
9m MCAO versus sham:	$p = 0.23$; $F(1) = 1.69$
20m MCAO versus sham:	$p = 0.042$; $F(1) = 4.816$

Velocity:

Day1

6m MCAO versus sham:	$p = 0.420$; $F(1) = 0.702$
7.5m MCAO versus sham:	$p = 0.477$; $F(1) = 0.543$
9m MCAO versus sham:	$p = 0.019$; $F(1) = 8.605$
20m MCAO versus sham:	$p = 0.008$; $F(1) = 8.948$

Day2

6m MCAO versus sham:	$p = 0.740$; $F(1) =0.116$
7.5m MCAO versus sham:	$p = 0.041$; $F(1) = 5.36$
9m MCAO versus sham:	$p = 0.253$; $F(1) = 1.515$
20m MCAO versus sham:	$p = 0.998$; $F(1) = 0001$

Day3

6m MCAO versus sham:	$p = 0.418$; $F(1) = 0.703$
7.5m MCAO versus sham:	$p = 0.001$; $F(1) = 38.767$
9m MCAO versus sham:	$p = 0.063$; $F(1) = 4.647$
20m MCAO versus sham:	$p = 0.547$; $F(1) = 0.377$

Day4

6m MCAO versus sham:	$p = 0.052$; $F(1) = 4.665$
7.5m MCAO versus sham:	$p = 0.011$; $F(1) = 9.224$
9m MCAO versus sham:	$p = 0.704$; $F(1) = 0.155$
20m MCAO versus sham:	$p = 0.206$; $F(1) = 1.708$

Day5

6m MCAO versus sham:	p = 0.316; F(1) = 1.113
7.5m MCAO versus sham:	p = 0.168; F(1) = 2.183
9m MCAO versus sham:	p = 0.871; F(1) = 0.028
20m MCAO versus sham:	p = 0.039; F(1) = 4.97

Water Maze probe trail data:

Statistical differences between sham and MCAO

NE-quadrant:

6m groups

Sham:	Mdn = 54 %;
MCAO:	Mdn = 35 %; U = 17.00; n = 14; p = 0.366;

7.5m groups

Sham:	Mdn = 58 %;
MCAO:	Mdn = 45 %; U = 13.00; n = 14; p = 0.156;

9m groups

Sham:	Mdn = 62 %;
MCAO:	Mdn = 36 %; U = 2.00; n = 11; p = 0.017;

20m groups

Sham:	Mdn = 32 %;
MCAO:	Mdn = 46 %; U = 49.00; n = 23; p = 0.321

SW-quadrant:

6m groups

Sham:	Mdn = 46 %;
MCAO:	Mdn = 51 %; U = 16.00; n = 12; p = 1.00;

7.5m groups

Sham:	Mdn = 57 %;
MCAO:	Mdn = 33 %; U = 3.00; n = 14; p = 0.005;

9m groups

Sham:	Mdn = 56 %;
MCAO:	Mdn = 38 %; U = 1.00; n = 11; p = 0.017;

20m groups

Sham:	Mdn = 35 %
MCAO:	Mdn = 38 %; U = 50.00; n = 23; p = 0.352

Water Maze hippocampus-dependent strategies:

Statistical differences between sham and MCAO

6 month

Day1:	p = 0.003, 95 % CI (1.828; 16.927)
Day2:	p < 0.001, 95 % CI (4.336; 208.13)
Day3:	p = 0.015, 95 % CI (0.13; 22.22)
Day4:	p = 0.715, 95 % CI (0.265; 2.533)

Day5:	p < 0.001, 95 % CI (0.19; 12.186)
7.5 month	
Day1:	p = 0.729, 95 % CI (0.348; 4.48)
Day2:	p = 0.008, 95 % CI (1.02; 9.737)
Day3:	p = 0.043, 95 % CI (1.042; 5.437)
Day4:	p = 0.003, 95 % CI (0.039; 0.612)
Day5:	p < 0.001, 95 % CI (16.03; 775.479)
9 month	
Day1:	p = 0.304, 95 % CI (0.103; 2.087)
Day2:	p = 0.602, 95 % CI (0.404; 4.10)
Day3:	p = 0.219, 95 % CI (0.586; 8.679)
Day4:	p = 0.515, 95 % CI (0.444; 4.416)
Day5:	p = 0.016, 95 % CI (1.223; 6.823)
20 month	
Day1:	p = 0.792, 95 % CI (0.499; 2.455)
Day2:	p = 0.734, 95 % CI (0.345; 2.105)
Day3:	p = 0.606, 95 % CI (0.331; 1.893)
Day4:	p = 0.530, 95 % CI (0.303; 1.818)
Day5:	p = 0.529, 95 % CI (0.652; 2.286)

Water Maze hippocampus-independent and -dependent strategies:

strategy 1	
group:	p = 0.022; df = 1; Chi_Square = 5.214
age:	p = 0.109; df = 3; Chi_Square = 6.050
day:	p < 0.001; df = 4; Chi_Square = 31.046
interaction between group and day:	p = 0.339; df = 4; Chi_Square = 4.532
strategy 2	
group:	p < 0.001; df = 1; Chi_Square = 23.173
age:	p = 0.009; df = 3; Chi_Square = 11.611
day:	p < 0.001; df = 4; Chi_Square = 92.262
interaction between group and day:	p = 0.002; df = 4; Chi_Square = 16.931
strategy 3	
group:	p = 0.513; df = 1; Chi_Square = 0.428
age:	p = 0.453; df = 3; Chi_Square = 2.628
day:	p < 0.001; df = 4; Chi_Square = 24.302
interaction between group and day:	p = 0.007; df = 4; Chi_Square = 14.159
strategy 4	
group:	p < 0.001; df = 1; Chi_Square = 33.291
age:	p = 0.096; df = 3; Chi_Square = 6.334
day:	p < 0.001; df = 4; Chi_Square = 220.117
interaction between group and day:	p < 0.001 ; df = 4; Chi_Square = 166.647
strategy 5	
group:	p = 0.001; df = 1; Chi_Square = 10.763
age:	p = 0.468; df = 3; Chi_Square = 2.541
day:	p < 0.001; df = 4; Chi_Square = 45.465
interaction between group and day:	p = 0.990; df = 4; Chi_Square = 0.297
strategy 6	

group: p = 0.087; df = 1; Chi_Square = 2.932
age: p = 0.925; df = 3; Chi_Square = 0.471
day: p < 0.001; df = 4; Chi_Square = 23.217
interaction between group and day: p = 0.094; df = 4; Chi_Square = 7.923

strategy 7

group: p = 0.101; df = 1; Chi_Square = 2.689
age: p = 0.067; df = 3; Chi_Square = 7.161
day: p < 0.001; df = 4; Chi_Square = 23.347
interaction between group and day: p = 0.082; df = 4; Chi_Square = 8.280

strategy 8

group: p = 0.799; df = 1; Chi_Square = 0.065
age: p = 0.516; df = 3; Chi_Square = 2.280
day: p < 0.001; df = 4; Chi_Square = 64.429
interaction between group and day: p = 0.005; df = 4; Chi_Square = 15.048

Water Maze hippocampus-independent and -dependent strategies per day:

Statistical differences between sham and MCAO

6 month-groups:

Day1

strategy 5: p = 0.022, 95 % CI (0.04; 0.776)

Day2

strategy 2: p = 0.001, 95 % CI (1.977; 12.332)
strategy 3: p < 0.001, 95 % CI (0.159; 1.244)
strategy 5: p = 0.008, 95 % CI (0.008; 0.481)

Day3

strategy 3: p = 0.001, 95 % CI (1.196; 10.661)
strategy 5: p = 0.001, 95 % CI (0.067; 0.969)

7.5 month-old groups:

Day1

strategy 2: p = 0.034, 95 % CI (0.217; 0.943)

Day2

strat2: p = 0.027, 95 % CI (1.149; 9.627)

9 month-old groups:

Day1

strategy 2: p = 0.042, 95 % CI (0.076; 0.953)
strategy 5: p = 0.041, 95 % CI (1.091; 77.780)

Day2

strategy 2: p = 0.005, 95 % CI (2.063; 30.579)

6-month-old groups:

strategy 3: p< 0.001, 95 % CI (1.716; 12.407)
strategy 7: p = 0.022, 95 % CI (0.058; 0.746)

7.5-month-old groups:

strategy 2: p = 0.016, 95 % CI (0.217; 0.943)
strategy 3: p = 0.019, 95 % CI (0.485; 4.629)
strategy 5: p = 0.012, 95 % CI (0.210; 2.432)
strategy 6: p = 0.036, 95 % CI (0.014; 0.866)

strategy 7:	p = 0.005, 95 % CI (0.010; 0.441)
9 month-old groups:	
strategy 2:	p = 0.029, 95 % CI (1.228; 69.112)
20-month-old groups:	
strategy 2:	p = 0.029, 95 % CI (1.430; 77.20)
